# Finerenone in heart failure with left ventricular ejection fraction ≥40%: a correspondence

**DOI:** 10.1097/MS9.0000000000003995

**Published:** 2025-09-30

**Authors:** Muhammad Furqan, Muhammad Usman Haider, Sadia Binte Rahim

**Affiliations:** a Department of Medicine, King Edward Medical University, Lahore, Pakistan; b Department of Internal Medicine, Geisinger Health System, Wilkes-Barre, PA, USA; c Department of Medicine, Mymensingh Medical College Hospital, Mymensingh, Bangladesh

**Keywords:** FINEARTS-HF, finerenone, hFmrEF, hFpEF, REDEFINE-HF

## Abstract

Finerenone, the first nonsteroidal mineralocorticoid receptor antagonist approved by the U.S. Food and Drug Administration for treating heart failure (HF) with mildly reduced ejection fraction and HF with preserved ejection fraction reduces morbidity and mortality by mitigating inflammation and fibrosis. The FINEARTS-HF (Finerenone in Heart Failure with Mildly Reduced or Preserved Ejection Fraction) trial demonstrated significant benefits, particularly after recent HF events, with ongoing studies evaluating broader applications across the cardio–kidney–metabolic spectrum.

The U.S. Food and Drug Administration (FDA) has approved finerenone for treating heart failure (HF) with mildly reduced ejection fraction (HfmrEF; EF 41%–49%), and HF with preserved ejection fraction (HFpEF; EF ≥50%)^[1,2]^. This approval establishes finerenone as the first and only FDA-approved nonsteroidal mineralocorticoid receptor (MR) antagonist indicated for these patients. MRs, expressed in cardiomyocytes and endothelial cells, are pathologically overactivated by aldosterone, promoting inflammation, fibrosis, and oxidative stress, while impairing the nitric oxide signaling pathway^[3]^. Finerenone acts by inhibiting these MRs, mitigating cardiac and vascular inflammation and fibrosis, and supporting the functional recovery^[4]^. The FINEARTS-HF (Finerenone in Heart Failure with Mildly Reduced or Preserved Ejection Fraction) trial is the basis for this approval.

The FINEARTS-HF Phase 3 trial that followed 6001 patients for a median of 32 months assessed the efficacy and safety of finerenone on morbidity and mortality in patients with HF and left ventricular ejection fraction (LVEF) ≥40%, and its results were published in September 2024 in *The New England Journal of Medicine*^[5]^. Finerenone significantly reduced the composite risk of worsening of HF and cardiovascular death (risk ratio [RR]: 0.84, 95% confidence interval (CI): 0.74–0.95). The greatest benefit was observed in patients enrolled soon after a recent HF event (within 7 days: RR: 0.74, 95% CI: 0.57–0.95; 7 days to 3 months: RR: 0.79, 95% CI: 0.64–0.97). In contrast, no clear benefit was seen in those enrolled more than 3 months after HF or without prior worsening (RR: 0.99, 95% CI: 0.81–1.21). The risk of adverse events such as hyperkalemia and renal events was not increased in patients with recent HF^[5]^.

Currently, a randomized, quadruple-blind, Phase 3 clinical trial, REDEFINE-HF, is also investigating the efficacy and safety of finerenone in reducing morbidity and mortality among patients hospitalized with HFmrEF or HfpEF^[6]^. The FINE-HEART pooled analysis also showed that finerenone reduced the risk of new-onset atrial fibrillation or atrial flutter across the cardio–kidney–metabolic (CKM) spectrum^[7]^. The American Heart Association defines CKM as the interconnected epidemic of obesity, type 2 diabetes, cardiovascular disease, and chronic kidney disease^[8]^. Hence, it can also be a groundbreaking therapeutic option to reduce the risk of atrial fibrillation or atrial flutter-related morbidity and mortality across the CKM spectrum^[7]^.

Data from the FDA Adverse Events Reporting System highlight an increased risk of acute kidney injury, hyperkalemia, and renal failure with finerenone. In the FINEARTS-HF, the most common adverse events in the finerenone group were anemia, unstable angina, acute kidney injury, hyperkalemia, and urinary tract infection^[5]^. Thus, initiation of finerenone in HF patients should be reserved for those with preserved renal function and a low risk of hyperkalemia, with continuous monitoring throughout therapy.

Diabetes mellitus, renal failure, and baseline renal insufficiency are known prognostic factors that worsen outcomes due to severe congestion and diuretic resistance^[9]^. Thus, with FDA approval, finerenone becomes the only drug that reduces cardiorenal risk by slowing kidney disease progression in type 2 diabetes while also lowering the risk of HF events in patients with LVEF ≥40%^[5,10]^. Depending on the results of the current research studies and ongoing trials, finerenone may be beneficial in reversing the remodeling of the heart and vasculature, thereby improving cardiac function. This can potentially redefine the management approach for patients with coexisting renal impairment and HFmrEF or HFpEF.

## Data Availability

The datasets are available from the corresponding author on reasonable request.

## Research registration unique identifying number (UIN)

This is not an original research project involving human participants in an interventional or an observational study but a case report. This registration was not required.

## AI use declaration

To ensure transparency, this work complies with the TITAN 2025 guidelines (Transparency In The reporting of Artificial INtelligence)^[11]^. All AI use has been declared and reviewed in line with these recommendations. Generative AI was employed only for grammar, spelling, and clarity improvements during drafting and revision, without involvement in data analysis or figure creation. Grammarly (web-based, version 14.1249.0) and ChatGPT (OpenAI GPT-5 via chat.openai.com, default settings) were used exclusively for language refinement, with no plug-ins, fine-tuning, or integrations. No patient text or images were used; all inputs complied with GDPR/HIPAA. Outputs were reviewed, edited, and approved by the supervising author who assumes full responsibility for the final content. AI tools were applied solely for language refinement, using simple text-based prompts, with full adherence to ethical standards.

## References

[1] FDA update: finerenone approved to treat HF patients with LVEF ≥40% - American College of Cardiology [cited July 19, 2025]. https://www.acc.org/Latest-in-Cardiology/Articles/2025/07/15/20/01/FDA-Update-Finerenone-Approved

[2] Andronic AA Mihaila S Cinteza M. Heart failure with mid-range ejection fraction – a new category of heart failure or still a gray zone. Maedica (Bucur) 2016;11:320–24. 28828050 PMC5543525

[3] Bauersachs J Lother A. Mineralocorticoid receptor activation and antagonism in cardiovascular disease: cellular and molecular mechanisms. Kidney Int Suppl 2022;12:19–26. 10.1016/j.kisu.2021.11.001 PMC9073241 35529088

[4] Holst-Hansen A Grimm D Wehland M. Finerenone in heart failure – a novel therapeutic approach. Int J Mol Sci 2024;25:13711. 39769473 10.3390/ijms252413711 PMC11678263

[5] Solomon SD McMurray JJV Vaduganathan M. Finerenone in heart failure with mildly reduced or preserved ejection fraction. N Engl J Med 2024;391:1475–85. 39225278 10.1056/NEJMoa2407107

[6] Colorado Prevention Center. Randomized trial to determine the efficacy and safety of finerenone on morbidity and mortality among heart failure patients with left ventricular ejection fraction greater than or equal to 40% hospitalized due to an episode of acute decompensated heart failure (REDEFINE-HF). clinicaltrials.gov; 2024 Feb [cited July 20, 2025]. Report No.: NCT06008197. https://clinicaltrials.gov/study/NCT06008197

[7] Pabon MA Filippatos G Claggett BL. Finerenone reduces new-onset atrial fibrillation across the spectrum of cardio-kidney-metabolic syndrome. JACC 2025;85:1649–60. 40306837 10.1016/j.jacc.2025.03.429

[8] Sebastian SA Padda I Johal G. Cardiovascular-Kidney-Metabolic (CKM) syndrome: a state-of-the-art review. Curr Probl Cardiol 2024;49:102344. 38103820 10.1016/j.cpcardiol.2023.102344

[9] Blair JEA Manuchehry A Chana A. Prognostic markers in heart failure--congestion, neurohormones, and the cardiorenal syndrome. Acute Card Care 2007;9:207–13. 17891672 10.1080/17482940701606913

[10] Filippatos G Anker SD Böhm M. A randomized controlled study of finerenone vs. eplerenone in patients with worsening chronic heart failure and diabetes mellitus and/or chronic kidney disease. Eur Heart J 2016;37:2105–14. 27130705 10.1093/eurheartj/ehw132 PMC4946749

[11] Riaz AA Ginimol M Rasha R. Transparency in the Reporting of Artificial Intelligence – The TITAN Guideline. Prem J Sci 2025;10:100082.

